# Is it Necessary to Stabilize Every Fracture in Patients with Serial Rib Fractures in Blunt Force Trauma?

**DOI:** 10.3389/fsurg.2022.845494

**Published:** 2022-06-09

**Authors:** Sebastian Reindl, Philipp Jawny, Evaldas Girdauskas, Stephan Raab

**Affiliations:** Department for Cardiothoracic Surgery, Medical Faculty, University Hospital Augsburg, Augsburg, Germany

**Keywords:** chest wall, blunt thoracic trauma, rib fractures, rib stabilization, flail chest, flail segment

## Abstract

**Introduction:**

Management of traumatic rib fractures is subject of controversial discussions. Rib fractures are common, especially after traffic accidents and falls. There is no consensus on whether and how many rib fractures need reconstruction. Not every rib fracture needs to be stabilized, but conservative treatment by internal splinting and analgesia is not effective for all patients. Deformities of the chest wall with reduced thoracic volume and restrictive ventilation disorders must be avoided. Intraoperative assessment of fractures and chest stability plays a central role.

**Material and methods:**

From 07/2016 to 07/2021, a total of 121 chest wall stabilizations were performed (m:f = 2:1, age 65 ± 14.5 a). Indications for surgery were the following criteria: (1) palpatory instability of the chest wall, (2) dislocated fracture endings, (3) concomitant injuries, (4) uncontrollable pain symptoms. In all patients, a computed tomography scan of the thorax was performed before the osteosynthetic treatment to assess dislocation of the fracture endings and possible concomitant injuries of intrathoracic organs.

**Results:**

Video-assisted thoracoscopy was performed in all patients. Hemothorax and concomitant injuries of the lung, diaphragm and mediastinum could be assessed. This was followed by an intraoperative assessment of the rib fractures, in particular penetration of fracture endings and resulting instability and deformity. Relevant fractures could be identified and subsequent incisions for rib osteosynthesis precisely defined. 6.3 (±2.7) rib fractures were detected, but 2.4 (±1.2) ribs treated osteosynthetically. Bilateral rib fractures were present in 26 patients (21.5%). Post-operative bleeding occurred in seven patients (5.8%), a breakage of the osteosynthetic material in two patients (1.7%).

**Discussion:**

Intraoperative assessment of relevant fractures and dislocation is the decisive criterium for osteosynthesis. Thoracoscopy is mandatory for this purpose – also to identify accompanying injuries. Not every fracture has to be approached osteosynthetically. Even with serial rib fractures or multiple fractures in a single rib, the thoracic contour can be restored by stabilizing only relevant fractures. Intraoperative palpation can adequately assess the stability and thus the result of the osteosynthesis. Even after surgical treatment of thoracic trauma, adequate analgesia and respiratory therapy are important to the healing process.

## Introduction

Thoracic trauma is responsible for a relevant number of deaths. In addition to the intrathoracic organs, the sequelae of injuries affect the bony chest wall in particular. In the absence of clear guidelines, therapeutic decisions and indications remain inconsistent when those injuries are detected.

The current annual report 2021 of the TraumaRegister DGU® (German Trauma Society) for the year 2020 (1) comprises 28,947 patients with thoracic truma (excluding slightly injured and survivors without intensive care unit therapy). The vast majority of these cases are blunt force trauma with a share of 96.3%. Penetrating injuries play a subordinate role in Germany. Relevant thoracic injuries (i. e. moderate injury severity, corresponding Abbreviated injury Score AIS ≥ 2) are found in almost half of these patients (45.4%). One of the most important therapies for these patients is a pleural drainage (prehospital 3.7%, intrahospital 10.5%). In a retrospective analysis of the registry data from 2009 to 2012, Schulz-Drost et al. were able to identify rib fractures in almost every 2nd patient in severely injured patients (ISS ≥ 16 points) (2). About a third of these were unstable serial rib fractures. In addition to this number of polytrauma patients, about 40,000 patients with rib fractures are added annually throughout Germany as a secondary diagnosis (3).

While there is consensus on the epidemiological significance, the clinical classification and documentation of bony thoracic injury (12 pairs of ribs and sternum) has so far been inconsistent. It was not until 2018 that the classification system of the AO Foundation (Arbeitsgemeinschaft für Osteosynthesefragen) / Orthopaedic Trauma Association (OTA) was extended to include a systematic of fractures of the thoracic wall (4).

**Figure 1:**
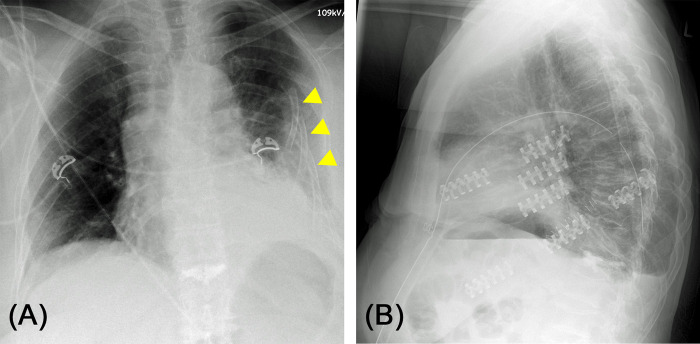
(**A**) Chest X-ray of a 73-year-old female patient after home accident. Significantly dislocated serial rib fracture of the left side with involvement of all ribs except the 12th rib. Several ribs with multiple fractures causing free fracture segments (“floating rib”). (**B**) Post-operative lateral chest X-ray after osteosynthesis of costae 4 to 7. Not all fractures needed to be fixated to achieve stabilization of the chest wall and regain physiological contour of the thorax.

Fractures of three or more consecutive ribs are defined as a serial rib fracture. Rib piece fractures lead to an instability of the thoracic wall, with the presence of two or multiple fractures of a rib with at least one free fracture segment (“floating rib”). An example is shown in Figure 1. If three or more ribs are affected, this is referred to as the so-called “flail chest”. If only one or two ribs are affected, this is referred to as a “flail segment” (5, 6). A fracture of the first rib is understood as a sign of severe thoracic trauma and suggests relevant injuries of large intrathoracic vessels or the brachial plexus (7, 8).

Analogous to the classification and definition, the therapeutic decision regarding rib serial or piece fractures is also inconsistent (9, 10). This is mainly due to the lack of greater cohorts of prospective randomized controlled trials. There are two metanalyses which indicate that patients with a flail chest profit from rib fixation (11, 12).

Conservatively, the concept of inner splinting is pursued along with enhanced analgesia (e.g., peridural catheter, intravenous analgesia or patient-controlled anaesthesia, PCA). Basically, the pain adjustment in the therapy of rib fractures is of eminent importance. In the unstable thorax, a paradoxical respiratory movement with reduced vital capacity and ineffective breathing in addition to lung contusion, hemato-/pneumothorax and pulmonary lacerations can result in pneumonia, which is a severe complication in these patients with increase in mortality and length of stay in hospital (2, 13, 14).

The S3-guideline “Polytrauma / Severely injured treatment” (2016, updated 2017) currently serves as a recommendation for the treatment of rib fractures (15). Conservative treatment by “internal pneumatic splinting in the context of overpressure ventilation” is favored. Surgical stabilization of the bony chest wall is recommended in hemodynamically stable patients. It is not to be approached as a matter of urgency in the case of severely injured patients.

In the guideline there is a suggestion for the indication of stabilizing rib fractures:

- unstable chest wall (flail chest) with weaning failure or paradoxical respiratory mobility during weaning
- prolonged pain
- chest wall deformity
- symptomatic pseudarthrosis
- thoracotomy for other indication (rib osteosynthesis on the “withdrawal” from the thorax)

The present retrospective analysis is intended to address the still unclear question which and how many ribs should be stabilized in individual cases.

## Patients and Methods

For the present study, all patients at Augsburg University Hospital with surgical chest wall stabilization from the time of accident to discharge from our acute inpatient care between 07/2016 and 07/2021 were retrospectively identified and evaluated. Patients with pseudarthrosis formation after rib fractures of any genesis were not included. A total of 121 chest wall stabilizations with osteosynthesis of rib fractures were performed in our department during the period mentioned.

The indication for surgical therapy was formed both from a clinical and radiological point of view:

### Clinical Indication

Polytraumatized patients were presented to us as part of the initial treatment in the trauma room, less seriously injured also in the routine treatment of our interdisciplinary emergency unit. Frequently, the first contact to the thoracic surgeon took place in the context of inpatient care at the nursery stations days after the accident occurred, often in the context of secondary trauma injuries such as persistent pneumothorax, hemorrhagic pleural effusion or secondary dislocation of rib fractures. In all patients, a careful physical examination was carried out after taking the patients or external anamnesis, in particular an inspection and palpation of the thoracic wall with assessment of any emphysema of the soft tissues, hematoma and rib fractures – especially regarding deformity and instability or paradoxical respiratory excursion in non-intubated patients.

Similar to the S3-guideline “Polytrauma / Severely injured treatment” (15), we see the following indications for rib stabilization:

- instability of the chest wall (“flail chest”, “flail segment”)
- dislocation of fracture endings of at least one width of a rib resulting in a deformity of the thoracic wall
- suspected / secured intrathoracic accompanying injuries due to rib fractures
- weaning problems (also pain-related)

### Radiological Indication and Preoperative Planning

All emergency trauma room patients shown in this analysis underwent a spiral computed tomography with multiplanar reconstruction, which was used for indication and preoperative planning of the therapeutic strategy for suspected concomitant injuries and the rib fractures to be stabilized. The remaining patients initially received a chest X-ray in two planes or a hemithorax imaging in two angulated projections as part of the initial treatment. As a result, in some cases the diagnosis of (serial) rib fracture(s) could already be made. For the definitive indication and surgical planning, however, we also demanded at least a computed tomography of the thorax in these patients (16).

**Figure 2:**
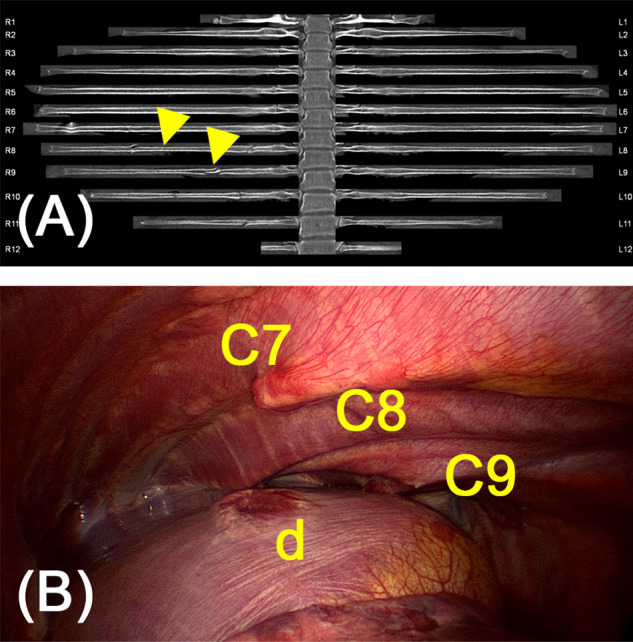
(**A**) Curvilinear reformations of all rib pairs of a 28-year old female patient after horse accident. Serial fracture of the 8th to 11th rib on the right dorsally with a dislocation by a maximum of one rib width of the 9th rib. In addition, fractures of the 7th and 8th rib on the right laterally are leading to flail chest. Arrows indicate flail segments of costae 7 and 8 due to multiple fracture. (**B**) Intraoperative assessment showed penetrating fracture endings and a lesion of the diaphragm (d) not detected by pre-operative diagnostics.

Multiplanar reconstructions make it possible to assess the bony thorax not only in the horizontal but also in coronal or sagittal planes. In selected patients, a 3-D volume rendering was also calculated from the data set of the CT examination (shown by example in Figure 7C), or a curvilinear reformation was carried out in order to be able to optimally assess the rib fractures (“unfolded ribs”, examples shown in Figures 2A, 8C) (17–19). In this way, the anatomically complex course of the ribs can be taken into account, and these can be viewed virtually in reconstructed two-dimensional layers. On the basis of these reconstructions the planning of the surgery is easier. Especially the question how many ribs and in case of multiple fractured ribs which fractures should be stabilized is more easily answered.

A specially defined algorithm for focused sonography (eFAST, extended Focused Assessment with Sonography for Trauma) enables the rapid detection of some acutely life-threatening accident consequences in the preclinical and clinical setting in trauma room patients (20). However, sonography is clinically of secondary importance for the indication of chest wall stabilization.

### Statistical Evaluation

Statistical calculations were performed using IBM SPSS Statistics, release 28 (IBM Inc, Armonk, New York, USA). For qualitative data, absolute and relative frequencies were calculated. Quantitative results were represented by their average or median together with the range. In order to compare two groups, student’s *t*-test or *χ*^2^ test were used as appropriate. Because of the rather small sample sizes, exact tests were performed.

The result of a statistical test was considered statistically significant if the *p* value was less than 0.05.

## Results

### Patient Characteristics

The male sex was predominant with a ratio of about 2:1 (male = 82, female = 39, *p* < 0.001). The median age of the patients was 65 ± 14.5 years. For the age-related assessment of rib fractures, the patients were also divided into age groups below and above 75 years of age:

- 83 patients <75 years
- 38 patients >75 years

The vast majority of patients (*n* = 111, 91.7%) had thoracic trauma after traffic accidents or falls, six patients had injuries after cardiopulmonary resuscitation, four patients had other causes (iatrogenic rib fractures). The proportion of polytrauma patients in the total population was 37.2% (*n *= 45) with an average ISS of 31.1 ± 9.9.

### Video-Thoracoscopic Assessment of Concomitant Injuries and Rib Fractures

Video-assisted thoracoscopy (VATS) was performed on all patients as part of the chest wall stabilization. Single lung ventilation was used for this purpose routinely. This initially relieved the hemothorax present in the majority of cases (*n* = 110, 83.5%). In this context, active sources of bleeding, especially the intercostal vessels, the internal thoracic artery and pericardial or diaphragm vessels, could also be identified and handled. If necessary for bleeding control, incisions for rib osteosynthesis could easily be extended to (mini-)thoracotomy. The assessment of accompanying intrathoracic injuries is also of great importance in this phase of trauma care: VATS has often been able to evaluate cryptogenic injuries of the lung parenchyma, the diaphragm and mediastinum, in particular (peri-) cardiac injuries. Especially diaphragmatic injury often escapes detection in preoperative imaging (see Figure 2).

For the final decision as to which and how many ribs need osteosynthesis, thoracoscopy was also used in all patients. As a result, it was possible to reliably identify fracture ends with dislocation and intrapleural skewering and to assess in detail which fractures caused instability or deformity by parallel palpation of the chest wall. This enabled an exact incision for open fracture reposition, also regarding the lowest possible soft tissue and surgical trauma.

### Number of Rib Fractures

In all analyzed patients, an average of 6.3 (±2.7) fractured ribs could be diagnosed on the basis of preoperative CT diagnostics. This number only assesses the presence of a fracture in the affected rib, but not multiple fractures in a single rib. A preference for either side of the main fractures could not be seen in our data (left = 60, right = 81).

Bilateral fractures were present in a total of *n* = 26 patients (21.5%) of these patients. On average, 5.1 (±2.9) contralateral rib fractures were detected by computed tomography. However, there was only an indication for bilateral stabilization in six of these cases (23.1% of cases with bilateral fractures, corresponding to 4.9% of all patients).

**Figure 3:**
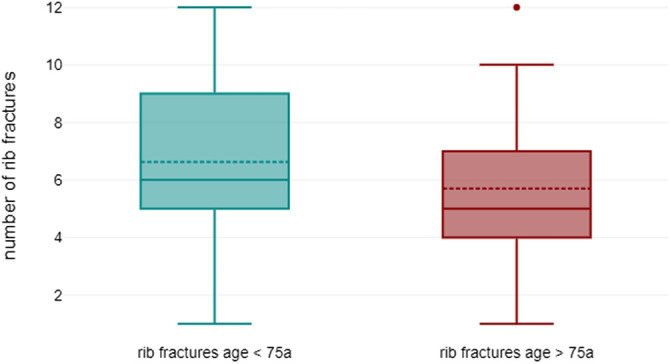
Comparison of rib fractures in younger (<75 years) and older patients (>75 years): mean 7.1 (±2.8) vs. 5.7 (±2.8) (*p* = 0.027).

Differences were revealed in a comparison of the age groups (see Figure 3): older patients above 75 years of age had a significantly lower number of rib fractures than younger patients below 75 years: mean 5.7 (±2.8) vs. 7.1 (±2.8). The difference was statistically significant (*p *= 0.027).

### Number of Stabilized Rib Fractures

**Figure 4:**
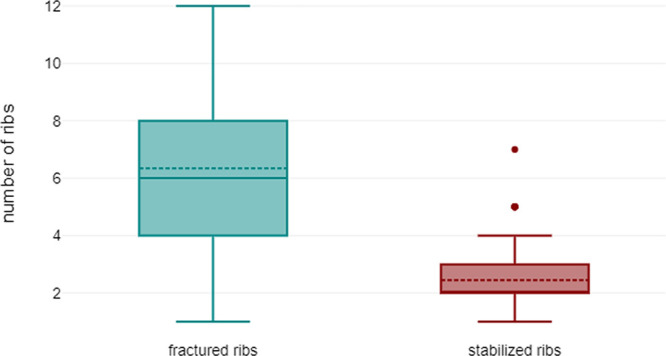
Comparison of fractured ribs vs. stabilized ribs in all patients: mean 6.3 (±2.7) vs. 2.4 (±1.2) (*p* < 0.001).

The number of fractures actually stabilized differed from that of fractured ribs: on average, 2.4 (±1.2) ribs had to be stabilized by osteosynthesis in order to achieve sufficient stability and compensate for any deformity of the chest wall. The difference between fractured and stabilized ribs was highly significant, as shown in Figure 4: *p* < 0.001, (95% confidence interval 3.461–4.354).

Decisive for this was the intraoperative assessment by the surgeon: by palpation of the chest wall and the video-thoracoscopic control of rib osteosynthesis. The following systems were used for osteosynthetic stabilization:

- StraCos Trauma: 113 (MedXpert, Eschbach, Germany)
- StraTos: 4 (MedXpert, Eschbach, Germany)
- MatrixRib and Titanium Sternal Fixation System: 6 (Johnson & Johnson Medical GmbH, Norderstedt, Germany)
- SternaLock Blu: 9 (Zimmer Biomet Germany GmbH, Freiburg, Germany)
- PDS cord: 12 (Johnson & Johnson Medical GmbH, Norderstedt, Germany)

The choice of osteosynthetic systems was based on intraoperative findings and localization (e. g. row fractures, parasternal fractures and injuries of the coastal arch). A comparison of both age groups revealed that significantly more rib fractures were stabilized in patients younger than in patients older than 75 years: mean 2.8 (±1.4) vs. 2.2 (±1.0) (*p* = 0.019).

### Stabilization of Only One Rib

**Figure 5:**
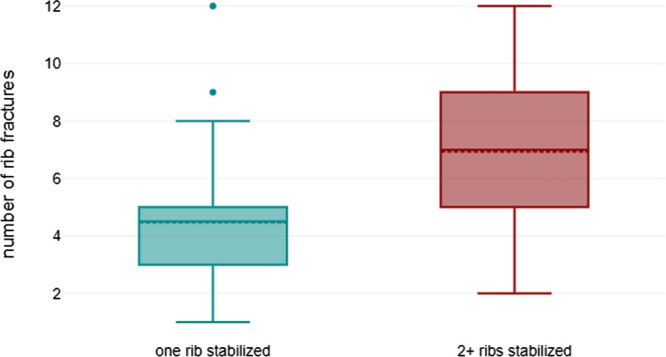
Comparison of the number of rib fractures present in patients undergoing osteosynthesis of a single to two and more ribs: mean 5.7 (±2.8) vs. 7.1 (±2.8) (*p* < 0.001).

In a relevant proportion of patients (*n* = 28, 23.1%), sufficient stability of the chest wall could be achieved by osteosynthesis of only a single rib fracture. In this subgroup, the average number of rib fractures was significantly lower with 4.5 (±2.6) in comparison to patients with two or more fixated ribs: 7.4 (±2.5) (*p* < 0.001, see Figure 5). In four of these patients, only a single rib was broken. Related to the age groups, this was possible in 17 younger patients (20.5%) and 11 elderly patients (28.9%).

### Complications and Outcome

**Figure 6:**
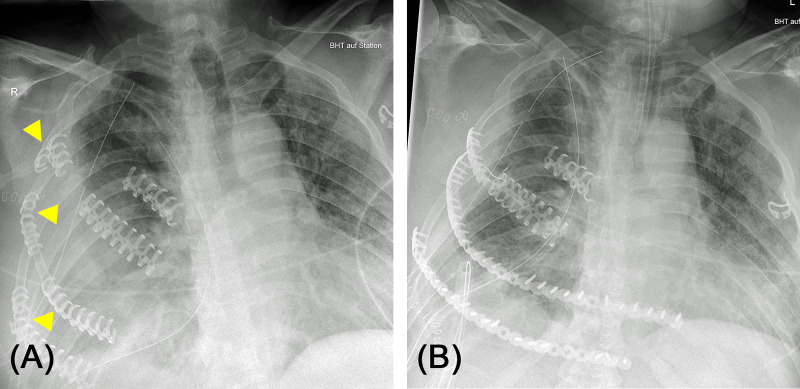
(**A**) 63-year-old male patient after car accident. Surgery for thoracic trauma was carried out at the day of hospital admission including rib fixation. The patient suffered from severe delirium afterwards. At day 7, chest X-ray revealed several broken osteosynthetic clips (arrows) causing recurrent instability of chest wall. (**B**) Post-operative chest X-ray after re-stabilization of the chest wall replacing broken clips with screwed titanium plates.

In a total of seven patients (5.8%), re-do intervention was indicated after surgical stabilization. Five of these patients experienced relevant post-operative bleeding which necessitated readmission to the operating room for removal of hematoma and hemostasis. Oral anticoagulation (phenprocoumon or direct oral anticoagulants) was present as a pre-medication in four of these patients and could not be discontinued beforehand due to the urgency of the procedure. Only two patients (1.7%) had fractures of osteosynthetic materials postoperatively, requiring re-osteosynthesis to be performed (see Figure 6). During the observation period, long-term ventilation with tracheotomy was indicated in a total of six patients (4.6%). A total of four patients died during acute inpatient treatment, all of whom were polytrauma patients with serious injuries resulting in multiple organ failure. Two patients had a relevant cardiac concomitant injury in the context of thoracic trauma.

The average length of acute inpatient treatment of 18.8 (±14.9, max. 94) days of the patients presented here is due to the time-consuming, interdisciplinary treatment of polytrauma patients. This is also confirmed by the average length of treatment of patients in intensive care units of 7.5 (±12.8, max. 76) days. Tendentially, the duration of treatment of older patients was shorter compared to the younger patients, but could not be proven statistically significant (*p* = 0.054).

## Discussion

### Criteria for Osteosynthesis of Rib Fractures

**Figure 7:**
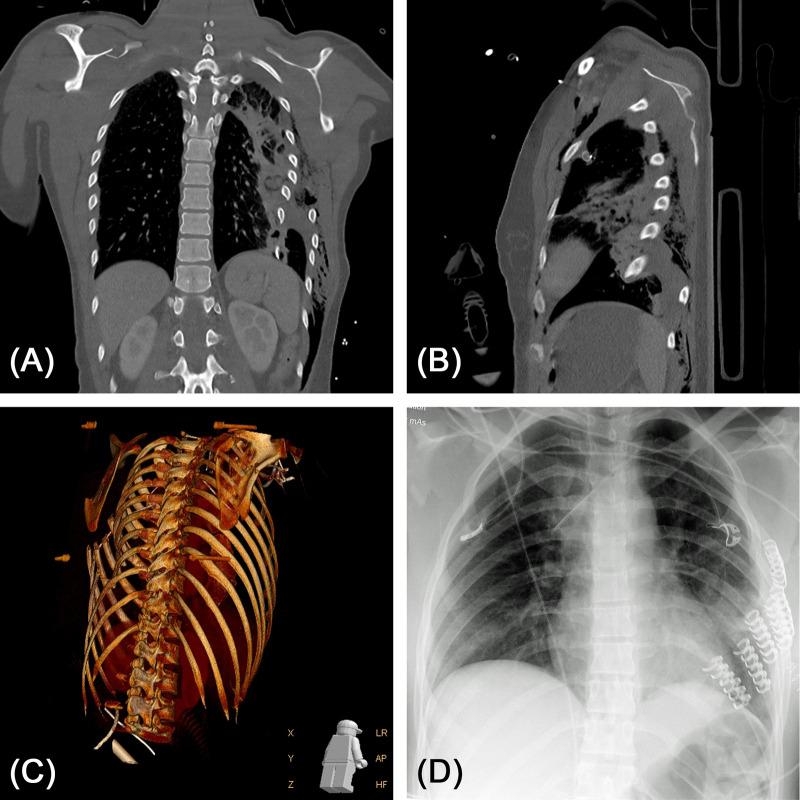
(**A,B**) 26-year-old female patient after suicidal train accident. Computed tomography (coronary and sagittal reconstructions are shown) revealed severely dislocated fractures of all ribs on the left side with laceration of the lung parenchyma and lung contusion. (**C**) 3D volume rendering of the CT dataset. (**D**) Post-operative chest X-ray after stabilization of costae 3 to 8. Laceration of the lung parenchyma was reconstructed by stapler-guided suture.

In principle, not every preoperatively proven fracture needs to be treated osteosynthetically (see Figure 7). This can be shown by the data of the study presented here: 6.3 (±2.7) rib fractures vs. 2.4 (±1.2) rib osteosyntheses. Thus, even in the presence of several fractures, a stable chest wall could be achieved by osteosynthesis of a single rib in almost a quarter of the patients.

In our experience, three criteria are decisive before one should consider the indication of osteosynthesis and the determination of the rib fractures to be treated:

1. physical examination by the surgeon to assess chest wall stability. This should be done both before the actual indication (trauma room, intensive care or nursery ward) and again immediately preoperatively in already analgo-sedated and relaxed patients.
2. preoperative diagnostics by CT scan of the thorax, if there is not already a CT trauma spiral. Thanks to reconstruction and reformation methods in radiology, rib fractures can be assessed in detail regarding the course of the fracture and dislocation as well as an existing deformity of the chest wall.
3. intraoperative assessment of fractures by VATS with assessment of stability by palpation. This assessment is done repeatedly after every stabilization until the chest wall is sufficiently stabilized and there is no deformity anymore.

In this context, a difference in the age groups was also remarkable: the older patient group (age >75 years) showed a significantly lower number of rib fractures compared to the younger patient group. In our experience, this reflects the low-energy and trivial trauma that are more likely in older age. At the same time, in this patient collective one must think of different injury patterns and an expectedly different spectrum of accompanying injuries (13). Regarding the role of fracture localization on the chest wall and associated intrathoracic injury, further analyzes are undoubtedly necessary.

### Intraoperative Assessment of Thoracic Wall Stability

**Figure 8:**
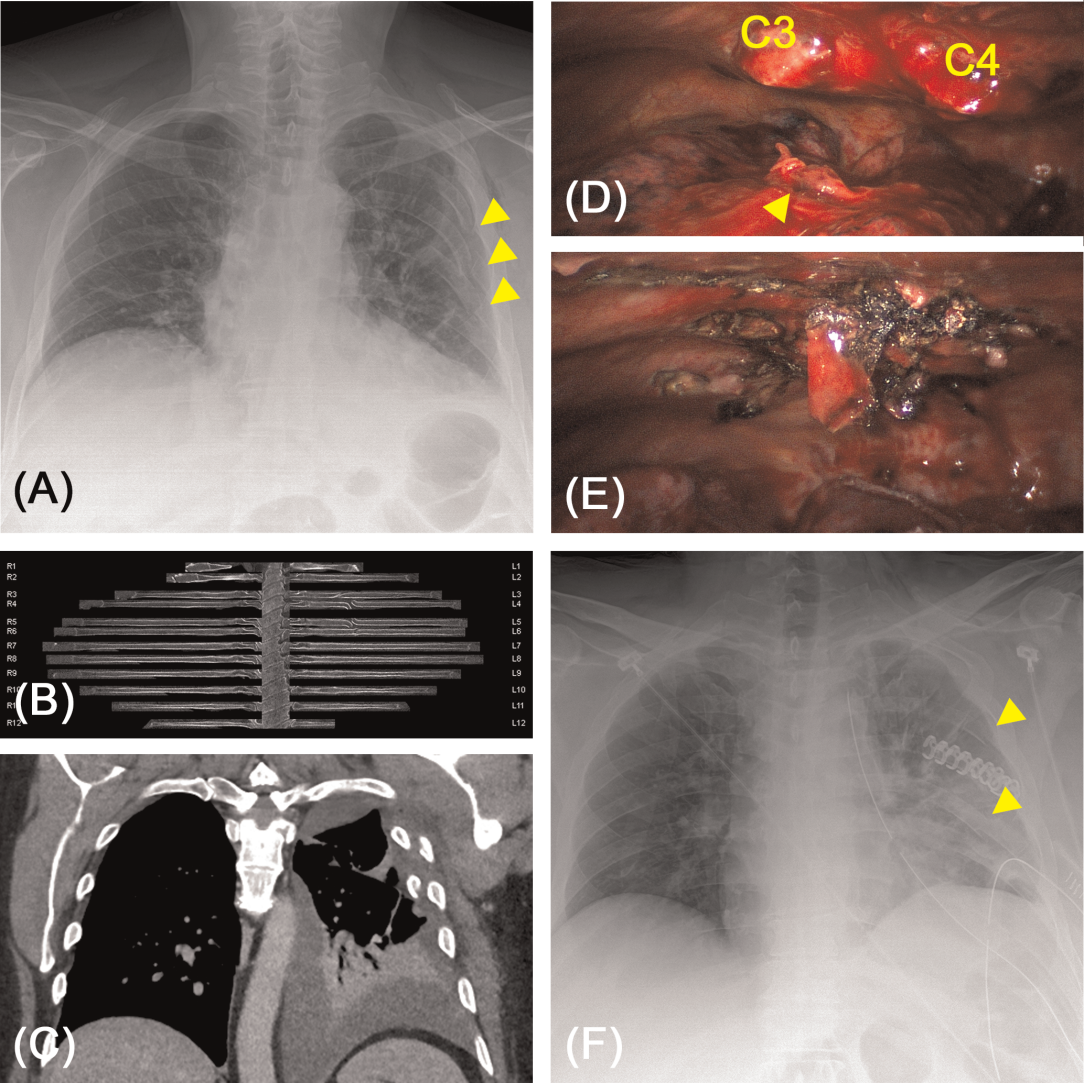
(**A**) 59-year-old-patient after fall. Sudden hemodynamic instability six days after the accident. Chest X-ray revealed secondary dislocation of rib fractures (arrows). (**B,C**) Computed tomography including curvilinear reformation confirmed the dislocation of fracture endings causing a novel hemothorax. (**D,E**) Intraoperative videoscopy showed dislocated fracture endings of costae 3 and 4 subscapular causing an actively bleeding laceration of the lung parenchyma (arrow). After osteosynthesis of costa 4 the lateral chest wall was sufficiently erected and palpatory stable. (**F**) Post-operative chest X-ray after rib stabilization, reconstruction of lung parenchyma and relieve of 800 mL hemothorax. By a single stabilization the adjacent ribs were also repositioned (arrows).

The adequate intraoperative assessment of thoracic wall stability by the surgeon is of particular importance. For this purpose, the video-thoracoscopic control of osteosynthesis regarding the thoracic contour and any remaining deformity as well as the synchronous palpatory examination of stability is crucial. In the case of serial fractures (“flail chest” or “flail segment” with “floating rib”), osteosynthesis of the ventral fracture is sufficient in most cases. Posterior and paravertebral rib fractures are sufficiently stabilized by autochthonous and dorsal muscles in most cases. Also, osteosynthesis causes high surgical trauma to access fractures in this localization. As a result, the flail chest or the flail segment can be converted into a simple, undislocated rib fracture. Figure 8 shows an example of this procedure which is based on the following considerations:

- minimizing the surgical trauma
- benefit from stabilization in patients with flail chest (11)

The complication dreaded most after rib fracture is pneumonia with and without pleural empyema. These patients are at risk for prolonged length of ICU and hospital stay, need for tracheostomy and have a higher mortality. Due to the flail chest and unstable thorax, poorly ventilated areas in the lungs result from paradoxical breathing (21). Due to the pain of a rib fracture without instability, insufficient ventilation can also occur. However, this can be remedied and prevented through adequate analgesia and physiotherapy (22).

In the case of physical instability with paradoxical movement, this is difficult or even impossible to achieve with conservative therapy. Therefore, our first goal in rib osteosynthesis is to restore the stability of the thorax and to correct deformities. Therapy for fractures that have not been treated is conservative with analgesia and physiotherapy. According to the study, the benefit of treating these remaining serial rib fractures is at least questionable. Our considerations and procedures are also supported by a study in which it was shown that complete stabilization of all ribs is not superior to partial stabilization (12).

Our procedure can thus be summarized as: minimize the surgical trauma, maximize the outcome.

### Time of Stabilization

The timing of surgical stabilization in patients with thoracic trauma also seems to have a relevant influence on the clinical outcome. A study published in 2020 by Otaka et al. reported significant reductions in the duration of mechanical ventilation, the duration of hospitalization and economic expenses, provided that the rib stabilization took place a maximum of 6 days after the accident occurred (23).

In our patient collective, fracture surgery took place on average 7.3 ± 7.1 days after the accident. Only eight patients required immediate surgery on the same day (6.6%).

### Conservative Therapy and Postoperative Care

The conservative care of singular, non-dislocated rib fractures is carried out with intensive analgesia and respiratory therapy. Stabilization is usually not necessary then. Apart from the immediate consequences of trauma, pneumonia occurs as one of the most common secondary complications. All efforts should be made to prevent this crucial complication.

An initially not completely relieved hemothorax (via effective pleural drainage) may later require a video-thoracoscopy. Without adequate treatment, patients are threatened by late complications, especially pleural empyema by superinfection. With unconsolidated rib fractures, painful pseudarthrosis can result in the long-term. Uncorrected deformities of the thoracic wall can also cause restrictive ventilation disorders with a reduction in vital capacity, FEV1 or diffusion capacity.

Serial rib fractures, an unstable thorax, dislocated fractures and accompanying injuries should therefore always be presented promptly to a thoracic surgeon or traumatologist with expertise in chest wall stabilization. Here, the timely and closely interlinked interdisciplinary care of trauma patients is of great importance.

### Limitation of the Study

A limitation of the presented as well as numerous other studies is the retrospective collection of the data and the restriction to patient collectives in which a chest wall stabilization was performed. Few randomized studies investigated – in comparatively small numbers of patients – the differences between surgical osteosynthesis and conservative therapy (24, 25). In this context, a reduced ventilation duration, a shorter intensive treatment and a lower risk of pneumonia are reported (26, 27). A significantly lower volume restriction, measured by the forced vital capacity and the total lung capacity, is also attributed to rib fixation (28, 29).

Meaningful prospective studies are urgently needed both regarding indication and postoperative outcome – especially pulmonary functional parameters.

## Data Availability

The original contributions presented in the study are included in the article/supplementary material, further inquiries can be directed to the corresponding author/s.

## References

[1] TraumaRegister DGU® (TR-DGU) Jahresbericht. (2021) Available from: https://www.traumaregister-dgu.de/fileadmin/user_upload/TR-DGU_annual_report_2021.pdf

[2] Schulz-Drost S Oppel P Grupp S Krinner S Langenbach A Lefering R Knöcherne Verletzung der Brustwand beim Polytrauma: Inzidenz, Begleitverletzungen, Verlauf und Outcome. Unfallchirurg. (2016) 119(12):1023–30. 10.1007/s00113-015-0026-7 26070732

[3] Schulz-Drost S Ekkernkamp A Stengel D. Epidemiology, injury entities and treatment practice for chest wall injuries: current scientific knowledge and treatment recommendations. Unfallchirurg. (2018) 121(8):605–14. 10.1007/s00113-018-0532-5 30073550

[4] Meinberg EG Agel J Roberts CS Karam MD Kellam JF. Fracture and dislocation classification compendium-2018. J Orthop Trauma. (2018) 32:S1–170. 10.1097/BOT.0000000000001063 29256945

[5] Lindenmaier HL Kuner EH Walz H. The surgical treatment of thoracic wall instability. Unfallchirurgie. (1990) 16(4):172–7. 10.1007/BF02588771 2219541

[6] Schelzig H Kick J Orend KH Sunder-Plassmann L. Thoraxverletzungen. Der Chirurg. (2006) 77(3):281–98. 10.1007/s00104-005-1146-3 16477430

[7] Richardson JD McElvein RB Trinkle JK. First rib fracture: a hallmark of severe trauma. Ann Surg. (1975) 181(3):251–4. 10.1097/00000658-197503000-00001 1130843 PMC1343844

[8] Sammy IA Chatha H Lecky F Bouamra O Fragoso-Iñiguez M Sattout A Are first rib fractures a marker for other life-threatening injuries in patients with major trauma? A cohort study of patients on the UK Trauma Audit and Research Network database. Emerg Med J. (2017) 34(4):205–11. 10.1136/emermed-2016-206077 28119351 PMC5502246

[9] Nirula R Diaz JJ Trunkey DD Mayberry JC. Rib fracture repair: indications, technical issues, and future directions. World J Surg. (2009) 33(1):14–22. 10.1007/s00268-008-9770-y 18949513

[10] Schuurmans J Goslings JC Schepers T. Operative management versus non-operative management of rib fractures in flail chest injuries: a systematic review. Eur J Trauma Emerg Surg. (2017) 43(2):163–8. 10.1007/s00068-016-0721-2 27572897 PMC5378742

[11] Leinicke JA Elmore L Freeman BD Colditz GA. Operative management of Rib fractures in the setting of flail chest: a systematic review and meta-analysis. Ann Surg. (2013) 258(6):914–21. 10.1097/SLA.0b013e3182895bb0 23511840 PMC3694995

[12] Beks RB Peek J de Jong MB Wessem KJP Öner CF Hietbrink F Fixation of flail chest or multiple rib fractures: current evidence and how to proceed. A systematic review and meta-analysis. Eur J Trauma Emerg Surg. (2019) 45(4):631–44. 10.1007/s00068-018-1020-x 30276722 PMC6689030

[13] Bergeron E Lavoie A Clas D Moore L Ratte S Tetreault S Elderly trauma patients with rib fractures are at greater risk of death and pneumonia. J Trauma. (2003) 54(3):478–85. 10.1097/01.TA.0000037095.83469.4C 12634526

[14] Athanassiadi K Theakos N Kalantzi N Gerazounis M. Prognostic factors in flail-chest patients. Eur J Cardio Thorac Surg. (2010) 38(4):466–71. 10.1016/j.ejcts.2010.02.034 20363148

[15] S3-Leitlinie Polytrauma / Schwerverletzten-Behandlung, AWMF Register-Nr. 012/019. (2016) Available from: https://www.awmf.org/uploads/tx_szleitlinien/012-019l_S3_Polytrauma_Schwerverletzten-Behandlung_2017-08.pdf

[16] Traub M Stevenson M McEvoy S Briggs G Lo SK Leibman S The use of chest computed tomography versus chest X-ray in patients with major blunt trauma. Injury. (2007) 38(1):43–7. 10.1016/j.injury.2006.07.006 17045268

[17] Pulley BR Taylor BC Fowler TT Dominguez N Trinh TQ. Utility of three-dimensional computed tomography for the surgical management of rib fractures. J Trauma Acute Care Surg. (2015) 78(3):530–4. 10.1097/TA.0000000000000563 25710423

[18] Ringl H Lazar M Töpker M Woitek R Prosch H Asenbaum U The ribs unfolded - a CT visualization algorithm for fast detection of rib fractures: effect on sensitivity and specificity in trauma patients. Eur Radiol. (2015) 25(7):1865–74. 10.1007/s00330-015-3598-2 25680714

[19] Khung S Masset P Duhamel A Faivre JB Flohr T Remy J Automated 3D rendering of ribs in 110 polytrauma patients: strengths and limitations. Acad Radiol. (2017) 24(2):146–52. 10.1016/j.acra.2016.09.018 27863898

[20] Schreyer C Schwab R. Management of thoracic trauma and intrathoracic injuries. Chirurg. (2020) 91(6):517–30. 10.1007/s00104-020-01176-w 32377762

[21] Caragounis EC Fagevik Olsén M Granhed H Rossi Norrlund R. CT-lung volume estimates in trauma patients undergoing stabilizing surgery for flail chest. Injury. (2019) 50(1):101–8. 10.1016/j.injury.2018.10.016 30482587

[22] Raab S Grieser T Sturm M Beyer M Reindl S. Management der Rippenfraktur. Zentralbl Chir. (2019) 144(3):305–21. 10.1055/a-0774-3401 31167271

[23] Otaka S Aso S Matsui H Fushimi K Yasunaga H. Early versus late rib fixation in patients with traumatic rib fractures: a nationwide study. Ann Thorac Surg. (2020) 110(3):988–92. 10.1016/j.athoracsur.2020.03.084 32360874

[24] Marasco SF Davies AR Cooper J Varma D Bennett V Nevill R Prospective randomized controlled trial of operative rib fixation in traumatic flail chest. J Am Coll Surg. (2013) 216(5):924–32. 10.1016/j.jamcollsurg.2012.12.024 23415550

[25] Tanaka H Yukioka T Yamaguti Y Shimizu S Goto H Matsuda H Surgical stabilization of internal pneumatic stabilization? A prospective randomized study of management of severe flail chest patients. J Trauma. (2002) 52(4):727–32. 11956391 10.1097/00005373-200204000-00020

[26] Iqbal HJ Alsousou J Shah S Jayatilaka L Scott S Scott S Early surgical stabilization of complex chest wall injuries improves short-term patient outcomes. J Bone Joint Surg Am. (2018) 100(15):1298–308. 10.2106/JBJS.17.01215 30063592

[27] Taylor BC Fowler TT French BG Dominguez N. Clinical outcomes of surgical stabilization of flail chest injury. J Am Acad Orthop Surg. (2016) 24(8):575–80. 10.5435/JAAOS-D-15-00476 27355282

[28] Granetzny A El-Aal MA Emam ER Shalaby A Boseila A. Surgical versus conservative treatment of flail chest. Evaluation of the pulmonary status. Interact Cardiovasc Thorac Surg. (2005) 4(6):583–7. 10.1510/icvts.2005.111807 17670487

[29] Lardinois D Krueger T Dusmet M Ghisletta N Gugger M Ris HB. Pulmonary function testing after operative stabilisation of the chest wall for flail chest. Eur J Cardiothorac Surg. (2001) 20(3):496–501. 10.1016/S1010-7940(01)00818-1 11509269

